# Disautonomias Pós-COVID: Importância do Reconhecimento Precoce e da Implementação de Programas de Recuperação

**DOI:** 10.36660/abc.20230110

**Published:** 2023-03-20

**Authors:** Denise Hachul, Tatiana Almeida, Mauricio Scanavacca

**Affiliations:** 1 Instituto do Coração HC FMUSP São Paulo SP Brasil Instituto do Coração - HC-FMUSP, São Paulo, SP – Brasil

**Keywords:** Pós-Covid/complicações, Disautonomias/pos-covid, Memória/deficiência, Fadiga, Depressão, SARS-COV-2, Reabilitação, Exercícios, Fisioterapia

A comunidade científica internacional dedicou todo o seu esforço nos últimos três anos, para o melhor entendimento e tratamento da infeção pelo SARS-COV-2, que inicialmente parecia limitar-se a uma síndrome respiratória aguda grave. No entanto, à medida que avançamos no tempo, deparamo-nos com um número crescente de pacientes com sintomas multissistêmicos, persistentes, intermitentes, ou tardios, que geraram importante comprometimento psico-funcional dos acometidos e que nos revelaram o caráter crônico e imprevisível da doença.^1^

A reconhecida Síndrome Pós-COVID, cursa com um número expressivo de queixas, descritas em relatos de casos, estudos populacionais e metanálises. Miocardites, infarto do miocárdio, acidente vascular cerebral, insuficiência cardíaca e arritmias, além de afecções genito-urinárias, gastrointestinais, endocrinológicas, neurológicas centrais e periféricas, de severidades variadas foram descritas na fase subaguda e crônica após contato com o vírus.^1-6^

Uma primeira série de casos foi publicada em pacientes italianos avaliados em média 60 dias depois da alta hospitalar. Os autores observaram que 87% apresentavam persistência de pelo menos um sintoma, 32% dois sintomas e 55% três ou mais. A fadiga foi a queixa mais frequente (53,1%), seguida de dispneia (43,4%), dor articular (27,3%) e dor torácica (21,7%).^2^

Em outro estudo, Huang et al., reportaram que 6 meses pós-alta, 76% dos indivíduos ainda apresentavam pelo menos um sintoma, especialmente fadiga, mialgia, artralgia, cefaleia, dispneia, dor torácica, palpitações, distúrbios do sono, deficiência de memória e da capacidade de concentração.^3^

Recentemente, três anos depois do início da pandemia, uma revisão detalhada publicada na *Nature Reviews Microbiology* estima que no mínimo 65 milhões de indivíduos ao redor do mundo mantenham-se sintomáticos e essa incidência continua aumentando, apesar do arrefecimento da infecção aguda, da menor mortalidade e do aumento de imunizados na população. Esse número pode ser ainda maior, considerando-se que muitos casos não são diagnosticados ou documentados na fase aguda da doença.^5^

A síndrome pós-COVID incide em todas as idades, mas predomina entre os 30 e 50 anos, com o número maior de acometidos está entre pacientes que tiveram infecção aguda leve. Esses dados refletem o padrão de distribuição epidemiológica da infecção na população mundial.^1-7^

Pesquisas sobre a qualidade de vida de pacientes com sintomas agudos leves e moderados apontaram que muitos evoluíram com distúrbios psiquiátricos, como depressão, ansiedade e síndrome do pânico. Quando comparados com pacientes internados, que necessitaram de oxigenioterapia ou ventilação mecânica, os últimos apresentaram três vezes mais queixas psiquiátricas, demostrando correlação traumática com a gravidade da doença aguda.^5,7^

Apesar do tempo decorrido entre os primeiros casos de infecção por SARS-COV-2 e os dias de hoje, a evolução e o significado dessas complicações tardias permanecem incertos.

A ação do SARS-COV-2 no sistema nervoso é altamente prevalente e seus mecanismos têm sido alvo de inúmeros estudos. Postula-se que o vírus atinja o sistema nervoso central, principalmente via receptores sensoriais olfativos e persista na região por longos períodos, causando alteração na velocidade da propagação dos impulsos elétricos das sinapses neurais, modificando os circuitos de controle central da dor, do humor e capacidade estratégica, além da coordenação geral e dos reflexos autonômicos. Alterações psico-cognitivas e autonômicas são as manifestações neurológicas mais prevalentes, podendo atingir 2/3 dos pacientes, que permanecem comprometidos por tempo indeterminado.^7-11^

Além do cérebro, a infecção por COVID-19 afeta o sistema nervoso autônomo por mecanismos diversos e complexos. A tempestade de citocinas pró-inflamatórias é acompanhada de uma hiperatividade simpática. A liberação exacerbada de catecolaminas causa taquicardia, dispneia, intolerância ortostática e aos esforços e dores no peito. Níveis altos de catecolaminas em ortostase prolongada ou após atividades físicas podem desencadear o reflexo de Bezold Jarisch ou vasovagal: uma supressão da atividade simpática e ativação parassimpática, causando vasodilatação paradoxal, hipotensão, e consequentemente, síncopes.^12-15^

Do ponto de vista cardiovascular, além da ação direta inflamatória do vírus no miocárdio e sistema neural intrínseco do coração, existem evidências de redução da volemia e do controle vasomotor, que também geram fadiga, mialgia, hipotensão e síncope. As síndromes da intolerância ortostática são exacerbadas pela hipovolemia, perda de massa muscular e do descondicionamento físico resultante. O repouso prolongado no leito colabora para a redução do volume sistólico, débito cardíaco e compromete a sensibilidade do barorreflexo.^5-7^

Estudos recentes observaram níveis baixos de cortisol no sangue de pacientes portadores de COVID longa, com mais de 1 ano de duração, quando comparados à população normal. A baixa produção de cortisol pela adrenal deveria ser compensada pelo aumento da produção de hormônio adrenocorticotrófico. Nesses pacientes, no entanto, não ocorre essa compensação, corroborando a existência de disfunção do eixo hipotálamo-hipófise-adrenal, possivelmente por acometimento global encefálico causado pelo virus.^14^

A disfunção autonômica relacionada à COVID-19 também pode ser mediada pela ação indireta do vírus. Na literatura já eram descritas inúmeras síndromes neurológicas imunomediadas como consequências de infecções virais e bacterianas.^16^A fadiga e a intolerância ortostática são manifestações subvalorizadas e estigmatizantes. As principais queixas dos pacientes são a redução da capacidade de realizar atividades cotidianas em relação ao período pré-infeccioso por períodos variáveis, acompanhadas de adinamia em repouso, fraqueza muscular aos pequenos esforços, sono não reparador, comprometimento cognitivo, taquicardias e intolerância ortostática. Outro sintoma frequente é a maior sensibilidade a estímulos sensoriais, cujo mecanismo é pouco conhecido.^7,10,16,17^

Há uma grande semelhança entre essas afecções pós-infeciosas já conhecidas e a síndrome Pós-COVID. O expressivo número de indivíduos infectados pelo SARSCOV-2 provocou um aumento substancial de pacientes com essas características, antes mais raramente observados e negligenciados, provocando um alerta para a comunidade científica sobre a importância do reconhecimento desses sintomas, extremamente limitantes.

A Síndrome da Taquicardia Postural Ortostática (*POTS*), muito frequente na Síndrome Pós-COVID, é caracterizada por sintomas de intolerância ortostática (na ausência de hipotensão significativa) e associados a aumento persistente da frequência cardíaca sinusal maior ou igual a 30 batimentos por minuto, quando o paciente permanece em pé por mais de 30 segundos, ou de pelo menos 40 batimentos por minuto em pacientes entre 12 e 19 anos de idade.^17,18^

Estudos de coorte já reportavam uma grande frequência de infecções prévias em pacientes que desenvolveram POTS e sua relação com biomarcadores autoimunes, como autoanticorpos anti α e β-adrenoceptores e anti-receptores muscarínicos. Pode-se então considerar que, além da ação viral inflamatória, existe um componente autoimune subjacente relacionado à síndrome pós-COVID. Tanto sintomas de POTS quanto de neuropatias periféricas de pequenas fibras são muito frequentes na síndrome pós-COVID. A síndrome de ativação mastocitária (SAM) também tem sido mais observada. Manifesta-se com erupções cutâneas associadas a prurido e cólicas abdominais. Estudos relatam aumento da ocorrência e maior gravidade dos sintomas de SAM em pacientes com COVID longa em comparação com os mesmos indivíduos pré-COVID e com grupos controle.^15-19^

Disautonomias são condições bastante desafiadoras. Os sintomas costumam ser diversos e de difícil correlação uns com os outros. Por isso, a maioria dos pacientes é interpretada como portadora de distúrbios psiquiátricos ou psicossomáticos. Essas condições, potencialmente disfuncionais, causam grande prejuízo na qualidade de vida, restrições sociais, impossibilidade de realização de atividade física, alimentando o ciclo vicioso e gerando cada vez maior incapacidade funcional. Essa incapacidade, por sua vez, desencadeia insegurança e, consequentemente, distúrbios psiquiátricos secundários.^20-26^

Por todos esses motivos, o reconhecimento precoce da síndrome Pós-COVID é de fundamental importância tanto para o seu portador, como para familiares, seu círculo profissional e social de convivência. Todos os indivíduos, após exposição ao SARS COV-2 e à imunização, que apresentem um ou mais sintomas relatados previamente, devem ser avaliados com atenção. A correlação do início dos sintomas com exposição ao vírus ou à vacina, mesmo que o paciente tenha sido oligo ou assintomático, é de extrema importância, já que sintomas da síndrome Pós-COVID podem se iniciar entre dias e semanas após o contato com o vírus e durar muitos meses.^20,25^

Um exame físico completo, cardiovascular, respiratório e neurológico é mandatório. Atenção especial deve ser dirigida ao estado nutricional, grau de hidratação, coloração de mucosas, frequência e padrão respiratórios, saturação de O_2_, presença de sarcopenia e acrocianose. Deve-se observar a postura, o equilíbrio, marcha, força muscular e cognição. Medidas da pressão arterial e da frequência cardíaca devem ser realizadas em decúbito dorsal, imediatamente após o paciente assumir a posição ortostática e depois de 3 minutos em pé para identificação de hipotensão ortostática e taquicardia postural inapropriada.^17,18,20,23^

Eletrocardiograma, laboratório bioquímico geral, provas de atividade inflamatória e exames de imagem são importantes na identificação de pneumonites, embolia pulmonar, alterações estruturais encefálicas e miocardiopatias estruturais. Monitorização com Holter, MAPA, dosagem de catecolaminas séricas fracionadas em DDH e em ortostase, de anticorpos anti-receptores muscarínicos e anti-adrenoceptores são indicados para confirmação de distúrbios autonómicos e identificação de sua fisiopatologia.^11,18,24,26^

O Teste de Inclinação (Tilt Table Test) é o método de escolha para diagnóstico de disautonomias neurogênicas e cardiovasculares. É um bom método quando o paciente não é diagnosticado durante exame físico em caso de suspeita e também para confirmação diagnóstica. Um protocolo ampliado - *Avaliação Autonômica*** - **deve ser realizado, com sistema de monitorização e aquisição de sinais contínuos e softwares especiais para medidas hemodinâmicas e cálculos da função autonômica completa (volume e índice sistólicos, débito e índice cardíacos, resistência vascular periférica, variabilidade do RR e a sensibilidade do Barorreflexo). Esses testes são importantes para identificação dos mecanismos da intolerância ortostática do paciente.^24,25^ O Doppler Transcraniano com medidas da velocidade do fluxo cerebral em DDH e ortostase pode demonstrar falhas dos mecanismos de autorregulação cerebrovascular, além de detectar alterações da resistência microvascular e obstruções dinâmicas ao fluxo sanguíneo.^26^

Escuta e acolhimento são os principais pilares do tratamento desses pacientes, na maioria das vezes estigmatizados e comprometidos por longos períodos. O apoio psicológico é de suma importância, especialmente e pela demora que experimentam para que o diagnóstico seja estabelecido. Devem ser feitas na primeira consulta orientações quanto a medidas comportamentais e dietéticas, higiene do sono e aconselhamento para evitar os gatilhos.

Corrigir a hipovolemia é a forma mais eficiente de evitar a hipotensão postural. É recomendada a hidratação de em média 2-3 litros de líquidos por dia, incluindo isotónicos e aumentando-se o sal da alimentação. Essas medidas garantem incremento do volume plasmático, além de melhorar a vasorreatividade periférica e o bombeamento vascular.^17,18^

Medicamentos hipotensores e diuréticos devem ser descontinuados se possível, assim como antidepressivos inibidores da recaptação da norepinefrina em casos de POTS.^17,18,24^Um programa regular de exercícios aeróbicos e de resistência muscular deve ser elaborado individualmente, depois de uma avaliação física detalhada. No início dos sintomas prodrômicos de baixo débito, o paciente pode realizar contrações musculares isométricas. Denominadas manobras de contrapressão muscular, têm como objetivo aumentar o retorno venoso.^17,18,26^

Se os sintomas persistirem apesar das medidas conservadoras, a terapia farmacológica deve ser considerada. A fludrocortisona, um expansor volêmico e alfa agonista, pode ser usada se a hipovolemia e baixa resistência periférica forem observadas na avaliação autonômica, como fatores fisiopatológicos. É um medicamento seguro, com mínimos efeitos colaterais e muito bem tolerado. Deve-se monitorar hipocalemia, incomum nas doses recomendadas. A midodrina, um agonista α-adrenérgico periférico, infelizmente não é comercializada no Brasil, mas pode ser adquirida por importação. O objetivo dessas medidas é melhorar o retorno venoso e a volemia central.^26^

Para pacientes hiperadrenérgicos, recomenda-se a clonidina e a metildopa, assim como betabloqueadores, preferencialmente com ação central. Em casos de comprovada neuropatia autonômica autoimune, o uso adjuvante de piridostigmina e imunoglobulina endovenosa pode ser indicado.^27,28^

Esses medicamentos prescritos individualmente ou em associação, permitem que o paciente se engaje em programas de reabilitação física e autonômica, que configura o principal tratamento para o resgate e impedimento de sequelas permanentes.^28,29^

O método que pode particularmente ser denominado de “*Reabilitação Autonômica*” leva em consideração as variáveis hemodinâmicas que contribuem para a melhora da sensibilidade do barorreflexo, do retorno venoso e, consequentemente, do débito cardíaco e pressão arterial em ortostase e durante atividades da vida diária, tanto em situações estáticas quanto dinâmicas.

Dormir com a cabeça da cama em aclive de 10 cm ajuda na adaptação vasomotora e diminui a perda volêmica noturna, minimizando hipotensões nos primeiros momentos de vigília. A inclinação do corpo, quando realizada repetidamente, equilibra o volume plasmático central. A ingesta hídrica e de sal (até 10 gramas por dia, salvo contraindicações), melhora a pressão arterial, a disposição e facilita a adesão à atividade física em tempo e qualidade. Recomenda-se o uso de meias elásticas de média compressão (com 30-40 mmHg) para minimizar o sequestro venoso periférico e aumentar o retorno venoso (idealmente aquelas que abranjam todo o membro inferior e o abdômen inferior) e tem seus efeitos otimizados principalmente com caminhadas diárias.^26-30^

A modulação da resposta autonômica mediada pelo exercício físico pode ser mensurada pela variabilidade da FC e níveis de catecolaminas plasmáticas.^30-32^ Esse reequilíbrio afeta positivamente o prognóstico das doenças cardiovasculares em geral.^33^ O treinamento físico aeróbio moderado e supervisionado, associado a exercícios de resistência muscular, promovem melhora na sensibilidade barorreflexa, com consequente aumento da tolerância ortostática. A ação do exercício sobre o sistema cardiovascular inclui aumento do volume de plasma circulante, aumento do tono e massa muscular e aprimora a redistribuição volêmica e a resposta hemodinâmica à posição ortostática. O efeito de “bomba muscular” promovido pela contração da musculatura esquelética das pernas auxilia no retorno venoso, impedindo o sequestro de sangue nas extremidades, aumentando a pressão de enchimento ventricular, o volume sistólico e o débito cardíaco. Além disso, é indiscutível seu papel na qualidade de vida e autoconfiança dos pacientes.

Apesar dos esforços e sistematizações do manejo não-farmacológico, a adesão às mudanças de hábitos e à realização de exercícios de pacientes com intolerância ortostática, depressão, dor crônica, sarcopenia e doenças neurológicas concomitantes, é um grande desafio. A hipotensão, fadiga, mialgia, dores articulares, dificuldade na coordenação motora e instabilidades do humor são os principais fatores limitantes para realização de exercícios domiciliares, já que os pacientes se tornam incapazes de controlar as intensidades de contração muscular e fazer ajustes posturais necessários para a sua eficácia. Por isso, no início, são imprescindíveis o acompanhamento presencial supervisionado e o tratamento concomitante das comorbidades associadas.^34^

Uma estratégia personalizada de reabilitação utilizando a estimulação sensório-motora interfere diretamente no eixo cérebro-coração e no balanço simpato-vagal do paciente. A estimulação vagal resulta em resposta anti-inflamatória e antagonista adrenérgica, sendo um alvo terapêutico promissor do sistema nervoso autônomo acometido nessas e em outras circunstâncias.^35,36^

Os primeiros relatos da ação da neuromodulação ou estimulação magnética transcraniana (TMS) nas funções autonômicas cardíacas foram observados em pacientes deprimidos, submetidos a estimulação cerebral repetitiva em regiões específicas cerebrais, especialmente o córtex pré-frontal dorsal esquerdo. A modulação autonômica do córtex temporal também promove redução da atividade simpática, aumento da sensibilidade dos barorreceptores e redução da frequência cardíaca no exercício submáximo. Essas intervenções influenciam diretamente o equilíbrio autonômico e neuroendócrino. Assim, a aplicação de TMS é capaz de atenuar o tônus simpático e aumentar o parassimpático, demostrando que o sistema cérebro-coração pode ser diretamente modulado. Ainda, a estimulação eletromagnética do córtex motor suplementar melhora a função do diafragma e a amplitude ventilatória em pacientes frágeis.^35-37^

A estimulação transcutânea do nervo vago (a nível do pavilhão auricular) representa uma outra oportunidade terapêutica para disautonomias e outras condições, por sua ação bidirecional na modulação autonômica, na reatividade cerebral e periférica e no sistema cardiovascular, interferindo especialmente na regulação da FC e na pressão arterial.^38-42^

Sua utilidade já está bem demonstrada no tratamento da depressão refratária e de dores crônicas, comorbidades frequentes em pacientes disautonômicos, com dificuldade de adesão para a prática de atividade física. Seus resultados foram validados pelo FDA. É importante ressaltar que critérios de segurança são imprescindíveis para a utilização da neuromodulação não invasiva, respeitando-se regulamentação para o uso deste recurso por profissionais de saúde treinados e capacitados.

Considerando-se as características dos disautonômicos, especialmente na síndrome Pós-COVID, a abordagem deve ser realizada por equipe multidisciplinar, com expertise nos métodos diagnósticos existentes e no manejo do paciente crônico. Esses pacientes, cujo número é crescente e imprevisível, têm condições médicas complexas e exigem cuidados integrativos. Serviços de atenção terciária com experiência nessa área são raros em nosso meio, gerando longos atrasos no diagnóstico e tratamento. A comunidade cardiológica deve estar atenta para o diagnóstico precoce e trabalhar para que programas especializados de recuperação sejam implementados, a fim de interromper a progressão da doença ou ao menos amenizar o desconforto dos pacientes.
