# Supplementary material for: Disautonomias Pós-COVID: Importância do Reconhecimento Precoce e da Implementação de Programas de Recuperação
Source: Arq Bras Cardiol. 2023 Mar 20;120(3):e20230110. [Article in Portuguese] doi: 10.36660/abc.20230110 (PMC10392843; doi:10.36660/abc.20230110)
Supplement: Translation

# Translation: Post-COVID Dysautonomias: The Importance of Early Recognition and Implementation of Recovery Programs

**Authors:** Denise Hachul, Tatiana Almeida, Mauricio Scanavacca

**Affiliations:** 1 Brazil Instituto do Coração - HC-FMUSP, São Paulo, SP – Brazil

**Keywords:** Post-Covid/complications, Dysautonomias/post-covid, Memory/deficiency, Depression, SARS-COV-2, Rehabilitation, Exercises, Physiotherapy

The international scientific community has devoted all its efforts over the last three years to a better understanding and treatment of the SARS-COV-2 infection, which initially seemed to be limited to a severe acute respiratory syndrome. However, as time goes by, we are faced with a growing number of patients with multisystemic, persistent, intermittent, or late symptoms, which generate significant psycho-functional impairment of those affected and reveal to us the chronic and unpredictable nature of the disease.^1^

The recognized Post-COVID Syndrome courses with a significant number of complaints described in case reports, population studies, and meta-analyses. Myocarditis, myocardial infarction, stroke, heart failure, arrhythmias, genitourinary, gastrointestinal, endocrinological, central, and peripheral neurological disorders of varying severities have been described in the subacute and chronic phases after contact with the virus.^1-6^

A first case series was published on Italian patients evaluated on average 60 days after hospital discharge. The authors noted that 87% had persistence of at least one symptom, 32% two symptoms, and 55% three or more. Fatigue was the most frequent complaint (53.1%), followed by dyspnea (43.4%), joint pain (27.3%), and chest pain (21.7%).^2^

In another study, Huang et al. reported that 6 months after discharge, 76% of individuals still had at least one symptom, especially fatigue, myalgia, arthralgia, headache, dyspnea, chest pain, palpitations, sleep disorders, impaired memory, and concentration capacity.^3^

Recently, three years after the start of the pandemic, a detailed review published in Nature Reviews Microbiology estimates that at least 65 million individuals around the world remain symptomatic, and this incidence continues to increase, despite the cooling of acute infection, lower mortality, and the increase in immunizations in the population. This number may be even higher, considering that many cases are not diagnosed or documented in the acute phase of the disease.^5^

Post-COVID syndrome affects all ages but predominates between 30 and 50 years old, and the highest number of patients affected are among those who had mild acute symptoms. These data reflect the epidemiological distribution pattern of the infection in the world population.^1-7^

Research on patients’ quality of patients who had mild or moderate acute symptoms revealed that many evolved with psychiatric disorders, such as depression, anxiety, and panic disorder. Compared to hospitalized patients who required oxygen therapy or mechanical ventilation, the latter had three times more psychiatric complaints, demonstrating a traumatic correlation with the severity of the acute illness.^5,7^

Despite the time elapsed between the first cases of SARS-COV-2 infection and the present day, the evolution and significance of these late complications remain uncertain.

The action of SARS-COV-2 on the nervous system is highly prevalent, and its mechanisms have been the subject of numerous studies. It is postulated that the virus reaches the central nervous system, mainly via olfactory sensory receptors, and persists in the region for long periods, causing changes in the speed propagation of electrical impulses in the neural synapses, modifying the central control circuits of pain, mood, and strategic capacity, in addition to general coordination and autonomic reflexes. Psycho-cognitive and autonomic alterations are the most prevalent neurological manifestations, reaching 2/3 of the patients who remain compromised for an indefinite period.^7-11^

In addition to the brain, COVID-19 infection affects the autonomic nervous system through diverse and complex mechanisms. The pro-inflammatory cytokine storm is accompanied by sympathetic hyperactivity. The exacerbated release of catecholamines causes tachycardia, dyspnea, and exertion intolerance, and chest pain. High levels of catecholamines in prolonged standing or after physical activities can trigger the Bezold Jarisch or vasovagal reflex: a suppression of sympathetic activity and parasympathetic activation, causing paradoxical vasodilation, hypotension, and consequently, syncope.^12-15^

From the cardiovascular point of view, in addition to the direct inflammatory action of the virus on the myocardium and the heart’s intrinsic neural system, there is evidence of a reduction in blood volume and vasomotor control, which also generate fatigue, myalgia, hypotension, and syncope. Orthostatic intolerance syndromes are exacerbated by hypovolemia, loss of muscle mass, and the resulting physical deconditioning. Prolonged bed rest contributes to the reduction of systolic volume and cardiac output and compromises baroreflex sensitivity.^5-7^

Recent studies have observed low cortisol levels in the blood of patients with long-term COVID, lasting more than 1 year, compared to the normal population. The increase in adrenocorticotropic hormone production should compensate for the adrenal’s low production of cortisol. In these patients, however, this compensation does not occur, corroborating the existence of hypothalamic-pituitary-adrenal axis dysfunction, possibly due to global encephalic involvement caused by the virus.^14^

Autonomic dysfunction related to COVID-19 may also be mediated through the indirect action of the virus. The literature has already described numerous immune-mediated neurological syndromes as consequences of viral and bacterial infections.^16^ Fatigue and orthostatic intolerance are undervalued and stigmatizing manifestations. The patients’ main complaints are reduced ability to perform daily activities compared to the pre-infectious period for variable periods, accompanied by adynamia at rest, muscle weakness on light exertion, unrefreshing sleep, cognitive impairment, tachycardia, and orthostatic intolerance. Another frequent symptom is greater sensitivity to sensory stimuli, the mechanism of which is little known.^7,10,16,17^

There is a great similarity between these already known post-infectious conditions and Post-COVID syndrome. The expressive number of individuals infected by SARSCOV-2 provoked a substantial increase in patients with these characteristics, previously more rarely observed and neglected, provoking an alert to the scientific community about the importance of recognizing these symptoms, which are extremely limiting.

Postural Orthostatic Tachycardia Syndrome (POTS), very common in Post-COVID Syndrome, is characterized by symptoms of orthostatic intolerance (in the absence of significant hypotension) and associated with a persistent increase in sinus heart rate greater than or equal to 30 beats per minute when the patient remains standing for more than 30 seconds, or at least 40 beats per minute in patients between 12 and 19 years of age.^17,18^

Cohort studies have already reported a high frequency of previous infections in patients who developed POTS and its relationship with autoimmune biomarkers, such as anti-α and β-adrenoceptor autoantibodies and anti-muscarinic receptors. It can then be considered that, in addition to the inflammatory viral action, there is an underlying autoimmune component related to the post-COVID syndrome. POTS and small peripheral fiber neuropathy symptoms are very common in post-COVID syndrome. Mast cell activation syndrome (MAS) has also been more frequently observed. It manifests with skin eruptions associated with itching and abdominal cramps. Studies report the increased occurrence and greater severity of MAS symptoms in patients with long-standing COVID compared to the same pre-COVID individuals and with control groups.^15-19^

Dysautonomias are quite challenging conditions. Symptoms are often diverse and difficult to correlate with each other. Therefore, most patients are interpreted as having psychiatric or psychosomatic disorders. These potentially dysfunctional conditions cause significant impairment in quality of life, social restrictions, and the impossibility of performing physical activity, feeding the vicious cycle and generating increasing functional disability. This inability, in turn, triggers insecurity and, consequently, secondary psychiatric disorders.^20-26^

For all these reasons, early recognition of the Post-COVID syndrome is of fundamental importance for the carrier and family members, as their professional and social circle. After exposure to SARS-COV-2 and immunization, all individuals who present with one or more previously reported symptoms should be carefully evaluated. The correlation between the onset of symptoms and exposure to the virus or vaccine, even in patients with mild acute symptoms or asymptomatic, is extremely important, as symptoms of Post-COVID syndrome can begin days and weeks after contact with the virus and last for many months.^20,25^

A complete physical examination of cardiovascular, respiratory, and neurological is mandatory. Special attention should be given to the nutritional status, degree of hydration, the color of mucous membranes, respiratory rate and pattern, O_2_ saturation, sarcopenia, and acrocyanosis. Posture, balance, gait, muscle strength, and cognition should be observed. Blood pressure and heart rate measurements should be performed in the supine position immediately after the patient assumes the orthostatic position, and after 3 minutes of standing, to identify orthostatic hypotension and inappropriate postural tachycardia.^17,18,20,23^

Electrocardiograms, general biochemical laboratory, tests of inflammatory activity, and imaging tests are important in identifying pneumonitis, pulmonary embolism, brain structural alterations, and structural cardiomyopathies. Monitoring with Holter, ABPM, measurement of fractionated serum catecholamines in supine and orthostasis, anti-muscarinic receptors, and anti-adrenoceptors antibodies are indicated for confirmation of autonomic disorders and identification of their pathophysiology.^11,18,24,26^

The Tilt Table Test is the method of choice for diagnosing neurogenic and cardiovascular dysautonomias. It is a good method when the patient is not diagnosed during physical examination in case of suspicion and for diagnostic confirmation. An expanded protocol - Autonomic Assessment - should be performed, with a monitoring system and acquisition of continuous signals and special software for hemodynamic measurements and calculations of complete autonomic function (systolic volume and index, cardiac output and index, peripheral vascular resistance, RR variability, and baroreflex sensitivity). These tests are important for identifying the patient’s orthostatic intolerance mechanisms.^24,25^ Transcranial Doppler with measurements of cerebral flow velocity in supine and orthostasis can demonstrate failures in cerebrovascular autoregulation mechanisms, in addition to detecting changes in microvascular resistance and dynamic obstructions to blood flow.^26^

Listening and welcoming are the main pillars of treating these patients, who are most often stigmatized and compromised for long periods. Psychological support is paramount, especially due to the delay they experience in the diagnosis to be established. The first appointment should provide guidance on behavioral and dietary measures, sleep hygiene, and counseling to avoid triggers.

Correcting hypovolemia is the most efficient way to prevent postural hypotension. Hydration of an average of 2-3 liters of liquid per day is recommended, including isotonic drinks and increasing the salt in the diet. These measures ensure an increase in plasma volume and improve peripheral vasoreactivity and vascular pumping.^17,18^

Hypotensive drugs and diuretics should be discontinued if possible, as well as norepinephrine reuptake inhibitor antidepressants in cases of POTS.^17,18,24^ A regular program of aerobic and muscular resistance exercises must be individually elaborated after a detailed physical evaluation. At the onset of low output prodromal symptoms, the patient can perform isometric muscle contractions. Called muscle counterpressure maneuvers, they aim to increase venous return.^17,18,26^

If symptoms persist despite conservative measures, pharmacologic therapy should be considered. Fludrocortisone, a volume expander and alpha agonist, can be used if hypovolemia and low peripheral resistance are noted on autonomic assessment as pathophysiological factors. It is a safe medication with minimal side effects and is very well tolerated. Monitor for hypokalemia, which is uncommon at recommended doses. Unfortunately, Midodrine, a peripheral α-adrenergic agonist, is not marketed in Brazil but can be purchased by import. The purpose of these measures is to improve venous return and central blood volume.^26^

For hyperadrenergic patients, clonidine and methyldopa are recommended, as well as beta-blockers, preferably with central action. In cases of proven autoimmune autonomic neuropathy, the adjuvant use of pyridostigmine and intravenous immunoglobulin may be indicated.^27,28^

These medications, prescribed individually or in combination, allow the patient to engage in physical and autonomic rehabilitation programs, which are the main treatment for rescuing and preventing permanent sequelae.^28,29^

The method that can be particularly called “Autonomic Rehabilitation” takes into account the hemodynamic variables that contribute to the improvement of baroreflex sensitivity, venous return, and, consequently, cardiac output and blood pressure in orthostasis and during activities of daily living in both static and dynamic positions.

Sleeping with the head of the bed at a 10 cm incline helps with vasomotor adaptation and decreases nocturnal volume loss, minimizing hypotension in the first moments of wakefulness. The body tilt, when performed repeatedly, balances the central plasma volume. Water and salt intake (up to 10 grams per day, unless contraindicated) improves blood pressure and disposition and facilitates adherence to physical activity in time and quality. The use of medium compression elastic stockings (with 30-40 mmHg) is recommended to minimize peripheral venous sequestration and increase venous return (ideally those covering the entire lower limb and lower abdomen), and its effects are optimized mainly with daily walks.^26-30^

The variability of HR and plasma catecholamine levels can measure the modulation of the autonomic response mediated by physical exercise.^30-32^ This rebalancing positively affects the prognosis of cardiovascular diseases in general.^33^ Moderate and supervised aerobic physical training, associated with muscular resistance exercises, promotes improved baroreflex sensitivity and consequent increase in orthostatic tolerance. The action of exercise on the cardiovascular system includes increased circulating plasma volume tone, and muscle mass and improves volume redistribution and hemodynamic response to the standing position. The “muscle pump” effect promoted by the legs’ skeletal muscles contraction helps venous return, preventing the sequestration of blood in the extremities and increasing ventricular filling pressure, systolic volume, and cardiac output. In addition, its role in patients’ quality of life and self-confidence is indisputable.

Despite the efforts and systematization of non-pharmacological management, adherence to changing habits and performing exercises in patients with orthostatic intolerance, depression, chronic pain, sarcopenia, and concomitant neurological diseases is a major challenge. Hypotension, fatigue, myalgia, joint pain, difficulty in motor coordination, and mood instabilities are the main limiting factors for performing home exercises, as patients become unable to control muscle contraction intensities and make necessary postural adjustments for its effectiveness. Therefore, supervised face-to-face monitoring and concomitant treatment of associated comorbidities are essential initially.^34^

A personalized rehabilitation strategy using sensorimotor stimulation directly interferes with the brain-heart axis and the patient’s sympathovagal balance. Vagal stimulation results in an anti-inflammatory and adrenergic antagonist response, being a promising therapeutic target for the affected autonomic nervous system in these and other circumstances.^35,36^

The first reports of the action of neuromodulation or transcranial magnetic stimulation (TMS) on cardiac autonomic functions were observed in depressed patients submitted to repetitive brain stimulation in specific brain regions, especially the left dorsal prefrontal cortex. Autonomic modulation of the temporal cortex also promotes a reduction in sympathetic activity, baroreceptor sensitivity increase, and heart rate reduction during submaximal exercise. These interventions directly influence the autonomic and neuroendocrine balance. Thus, applying TMS can attenuate the sympathetic tone and increase the parasympathetic tone, demonstrating that the heart-brain system can be directly modulated. Furthermore, electromagnetic stimulation of the supplementary motor cortex improves diaphragm function and ventilatory amplitude in frail patients.^35-37^

Transcutaneous stimulation of the vagus nerve (at auricular region) represents another therapeutic opportunity for dysautonomia and other conditions due to its bidirectional action on autonomic modulation, cerebral and peripheral reactivity, and the cardiovascular system, interfering especially in the regulation of HR and blood pressure.^38-42^

Its usefulness has already been well demonstrated in treating refractory depression and chronic pain, frequent comorbidities in dysautonomic patients with difficulty in adhering to the practice of physical activity. The FDA has validated its results. It is important to emphasize that safety criteria are essential for using non-invasive neuromodulation, respecting regulations for using this resource by trained and qualified health professionals.

Considering the characteristics of dysautonomia, especially in Post-COVID syndrome, the approach should be carried out by a multidisciplinary team with expertise in existing diagnostic methods and the management of chronic patients. These patients, whose numbers are growing and unpredictable, have complex medical conditions and require integrative care. Tertiary care services with experience in this area are rare, generating long delays in diagnosis and treatment. The cardiology community must pay attention to early diagnosis and work, so that specialized recovery programs are implemented to stop the progression of the disease or at least alleviate the discomfort of patients.
